# Fetal cord blood and tissue immune responses to chronic placental inflammation and chorioamnionitis

**DOI:** 10.1186/s13223-018-0297-y

**Published:** 2018-11-19

**Authors:** Anne Marie Singh, Michael G. Sherenian, Kwang-Youn Kim, Kristin A. Erickson, Amy Yang, Karen Mestan, Linda M. Ernst, Rajesh Kumar

**Affiliations:** 1 Division of Allergy and Immunology, Department of Pediatrics, Northwestern Feinberg School of Medicine, Ann and Robert H. Lurie Children’s Hospital of Chicago, 255 E Chicago Ave, Box #60, Chicago, IL 60611 USA; 2 Department of Preventive Medicine, Northwestern University Feinberg School of Medicine, Chicago, IL USA; 3 Biostatistics Collaboration Center, Northwestern University Feinberg School of Medicine, Chicago, IL USA; 4 Division of Neonatology, Ann and Robert H. Lurie Children’s Hospital of Chicago, Chicago, IL USA; 5 Department of Pathology and Laboratory Medicine, Northshore University Health System, Evanston, IL USA; 6 Department of Medicine, Northwestern Feinberg School of Medicine, Chicago, IL USA

**Keywords:** Chorioamnionitis, Asthma, Pediatrics, T cells, Foxp3, RORγt

## Abstract

**Background:**

Chorioamnionitis is a risk factor for future asthma development. Animal models of chorioamnionitis demonstrate increased T_H_17-to-T_reg_ ratios associated with proinflammatory cytokine elevations. The association of chorioamnionitis on human neonatal immune cells systemically and within tissues is not known.

**Methods:**

We enrolled two cohorts to evaluate T_H_17 and regulatory T cell (T_reg_) phenotypic markers in chorioamnionitis. From a cohort of 19 live birth infants, we collected cord blood and placenta samples to evaluate for signs of acute and chronic histologic inflammation and cell phenotype characterization. We analyzed a second cohort of stillborn infants with and without chorioamnionitis to classify and enumerate cell infiltrate phenotypes in the spleen, thymus, and lung. We used linear regression analysis determine the association of retinoic acid-related orphan receptor gamma t positive (RORγt^+^) and T_reg_ cell frequency with different types of inflammation seen in the live cohort subjects. Using linear mixed models, we evaluated for any associations between chorioamnionitis and T- and B-cell with a logarithmic scale for level of expression of cellular markers. We then performed Wilcoxon rank sum tests to assess the associations between cell count and chorioamnionitis.

**Results:**

In the live birth subjects with chronic placental inflammation we observed an increased proportion of RORγt^+^ cells in Foxp3^+^ cells, regardless of the presence of acute inflammation, compared to subjects with neither acute nor chronic inflammation. We also found an increased proportion of RORγt^+^ cells within Foxp3^+^ cells in subjects with acute high stage fetal and maternal inflammation compared to those without acute or chronic inflammation. In the stillborn subjects with chorioamnionitis, we observed a decrease in splenic Foxp3^+^ cells and an increase in lung CD3^+^ cells compared with subjects that did not have chorioamnionitis.

**Conclusion:**

Exposure to chorioamnionitis in utero may affect immune activation in neonates with an increased frequency of RORγt^+^ cells systemically as well as lymphocytic infiltrate in the lung. Our findings suggest an increase in RORγt^+^ cells during chorioamnionitis and thus may support the known associations between chorioamnionitis with asthma.

**Electronic supplementary material:**

The online version of this article (10.1186/s13223-018-0297-y) contains supplementary material, which is available to authorized users.

## Background

Factors affecting lung growth or lung function in fetal life may have a long-term effect on lung disease later in life [1, 2]. Histologic chorioamnionitis, a common inflammation of the maternal–fetal interface, complicates 8% of pregnancies, and is present in more than 50% of preterm births [3]. Previously, we, and others, demonstrated an association between chorioamnionitis and wheezing/asthma, decreased lung function, and other respiratory morbidities [1, 2, 4–8]. However, the mechanisms by which chorioamnionitis leads to wheezing, asthma or altered lung function are not well understood.

Chorioamnionitis may promote an inflammatory state in neonate [9]. Indeed, it has been shown that cord blood levels of interleukin (IL) 1β and IL-6 increase in neonates with chorioamnionitis. IL-6 exposure may then alter T-cell development to promote development of T-helper-cell 17 (T_H_17) effector responses and may also repress regulatory T-cell (T_reg_) development. The “master regulator” of Th17 cells is retinoic acid-related orphan receptor gamma (RORγt), which directs the development of Th17 cells [10]. These cells have been found to mediate inflammation, autoimmune disease, and may also protect from extracellular pathogens [11]. Regulatory T cells are diverse set of T cells that maintain immune homeostasis, tolerance and limit inflammation. FOXP3 has been identified as the master regulator of Treg development [12, 13]. It is suspected that T_reg_ and T_H_17 cells may be reciprocally regulated, and IL-17+ like Tregs have been described. These IL-17+ T_reg_ cells differentiate to inflammatory T_H_17 ex vivo, with a loss of FOXP3 expression [14]. Further complicating the issue, these cells have also been found to be immunosuppressive in the gut, likely promoting tolerance to extracellular microbes [15, 16]. Thus, the changes in cytokine expression in neonates with chorioamnionitis may eventually promote immune dysregulation, airway remodeling via mucous cell metaplasia, smooth muscle proliferation/migration, and further T_H_17 activation. Furthermore, animal models of chorioamnionitis demonstrate an increased lung and splenic T_H_17-to-T_reg_ ratio, which establishes a proinflammatory state [17–19]. However, the effects of chorioamnionitis on human fetal immune deviation and T-cell subsets have not been established. We hypothesized that, in human fetuses, chorioamnionitis is associated with systemic differences in regulatory T cells and T_H_17 cell number. We also hypothesized that there would be tissue specific differences in regulatory T cell profiles in the tissues. To evaluate these hypotheses, we examined two cohorts. We first prospectively investigated cord blood from 19 infants with clinical chorioamnionitis for T-cell marker expression. Second, we sought to determine any tissue specific alterations by evaluating a retrospective cohort of 20 still births of varying gestational age with and without chorioamnionitis.

## Methods

### Live birth cohort

Nineteen live preterm neonates delivered between 28 and 35 gestational weeks were enrolled. We obtained cord blood and placental samples from all enrolled infants under an institutional review board approved protocol. We performed hematoxylin and eosin (H&E) staining on all placental samples to assess for the presence of acute and chronic inflammation. We defined acute histologic chorioamnionitis as the presence of any marginating neutrophils in the extraplacental membranes, chorionic plate or umbilical cord. We then divided inflammation into maternal and/or fetal acute inflammatory responses. We assessed and staged the maternal acute inflammatory response in the membranes and chorionic plate as follows: Stage 1 for acute subchorionitis and chorionitis; Stage 2 for acute chorioamnionitis; Stage 3 for necrotizing acute chorioamnionitis. We considered Stage 2 or 3 maternal acute inflammation as high staging. We then classified maternal high stage subjects as having maternal evidence of acute chorioamnionitis for our analysis. We assessed and staged fetal acute inflammatory response in the chorionic plate vessels or umbilical cord as follows: Stage 1 for acute umbilical phlebitis or acute chorionic vasculitis; Stage 2 for acute umbilical arteritis, Stage 3 for acute necrotizing funisitis. We considered Stage 2 or 3 fetal acute inflammation as high stage. We then classified fetal high stage subjects on the presence of evidence for fetal acute chorioamnionitis for our analysis.

We also delineated the presence of chronic placental inflammation, which was characterized when we observed chronic inflammatory cells (lymphocytes, histiocytes, or plasma cells) in the membranes (chronic chorioamnionitis, chronic choriodeciduitis), basal plate (chronic deciduitis with plasma cells, chronic decidual perivasculitis), or villi (low or high grade chronic villitis, chronic intervillositis).

We recruited 12 preterm neonates with histologic chorioamnionitis and seven gestational age-matched controls without clinical chorioamnionitis. We present the characteristics of the recruited subjects, stratified by the presence of clinical chorioamnionitis, in Table 1. For the purposes of the analyses, we used histologic (not clinical) criteria to classify subjects. Of the 19 subjects, seven placentas had no histologic evidence of acute or chronic inflammation. Of the remaining 12 subjects, two subjects had maternal and fetal high stage acute inflammation in addition to chronic inflammation, one subject had maternal high stage acute inflammation as well as chronic inflammation, one subject had maternal high stage acute inflammation but no chronic inflammation, and eight subjects had only chronic inflammation (Additional file 1: Figure S1).

**Table 1:** Demographic data for subjects that underwent cellular, cord blood, analyses

	Chorioamnionitis (n = 12)	Control (n = 7)
Maternal age (years)		
Mean (range)	33 (18–47)	29 (20–37)
Median (interquartile range)	31 (27–41)	29 (28–31)
Maternal race		
White	4	1
Black	6	6
Other	2	0
Maternal ethnicity		
Hispanic	2	0
Non-Hispanic	9	7
Declined	1	0
Infant sex (male)	6	3
Gestational age (weeks)		
Mean (range)	31 (25–33)	32 (29–35)
Median (interquartile Range)	32 (31–33)	33 (32–34)
Preterm labor (yes)	9	5
Premature rupture of membranes (yes)	7	3
Membrane rupture		
Artificial	3	3
Spontaneous	9	4
Mode of delivery		
Vaginal	8	5
Cesarean	4	2
Multiple gestation (yes)	5	3
Antenatal steroids (yes)	6	4
Apgar at 1 min (average, range)	5 (1–9)	7 (3–9)
Apgar at 5 min (average, range)	8 (7–9)	8 (8–9)
Birthweight (grams)		
Mean (range)	1821 (740–2525)	1939 (1220–2670)
Median (interquartile range)	1882 (1734–1996)	1855 (1625–2290)

**Fig. 1:**
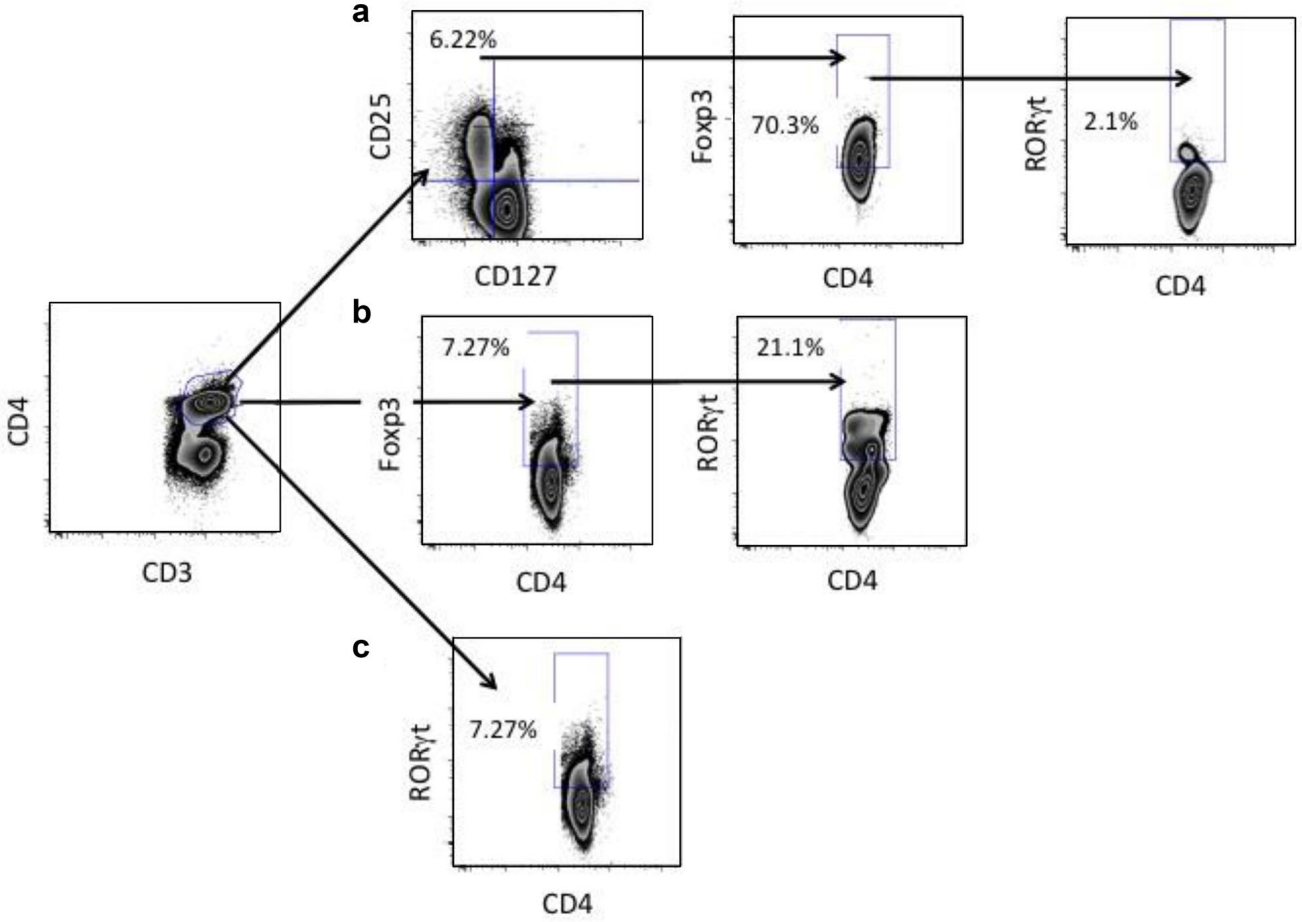
Flow cytometry gating strategy for cord blood analysis and percentage of each population selected. For full details refer to “Methods” section within the text. **a** CD25^+^CD127^−^ cells were gated from CD3^+^CD4^+^ cells. Foxp3^+^CD4^+^ cells were then gated from the CD25^+^CD127^−^ cells. Lastly RORγt^+^CD4^+^ cells were selected from the Foxp3^+^CD4^+^ cell population. **b** Foxp3^+^CD4^+^ cells were selected from the initial CD3^+^CD4^+^ population. These were then gated to select RORγt^+^CD4^+^ cells. **c** The initial CD3^+^CD4^+^ cell population was gated to select RORγt^+^CD4^+^ cells

To determine cord blood responses, we prospectively evaluated the T-cell phenotypes of the neonate via flow cytometry of cord blood from live preterm infants with and without chorioamnionitis. We evaluated cord blood mononuclear cells from the 19 neonates for surface and intracellular markers of T_reg_ and T_H_17 cells. We collected approximately 0.5–1 mL of cord blood from study participants in K2 EDTA tubes. We processed almost all samples immediately upon arrival, one patient was processed at approximately 24 h. (Thus, all samples were processed within 30 h of collection.) For cord blood cell isolation, we used Ficoll gradient separation, washed the cells with PBS (sterile Dulbeccos’ Phosphate buffered saline) and standard T-cell media, counted the cells and placed into flow tubes at 1 × 10^6^ cells/100 µL. We used the remaining cells for Fluorescence Minus One (FMO) controls. We then immediately stained the cells for flow cytometry analysis. We stained the cells for surface markers using the following antibody markers from the indicated source: CD3 APC-CY7 (SK7)-Biolegend, CD4V-500 (RPA-T4) BD, CD25 APC (BC96)-Biolegend, CD127 Pacific Blue (A019D5)-Biolegend. We then washed, fixed, and permeabilized the cells for intracellular staining with Forkhead Box P3 (Foxp3) Alexa Fluor 488 (206D)-Biolegend and retinoic acid-related orphan receptor gamma t (RORγt) PE (AKFJS-9) eBioscience. We performed acquisition using an LSR-II (BD, San Jose, CA) and analyzed using FlowJo software (Treestar, San Carlos, CA). We present the gating strategies for our flow analysis in Fig. 1.

We defined regulatory T-cells as CD3^+^CD4^+^CD25^+^CD127^lo^Foxp3^+^ cells within the lymphocyte gate. We examined expression of RORγt within CD3^+^CD4^+^ T-cells, CD3^+^CD4^+^Foxp3^+^ cells and CD3^+^CD4^+^CD25^+^CD127^lo^Foxp3^+^ cells. We express cell data as the median percentage of cells within either the gated population of CD3^+^CD4^+^ T-cells or the gated population of CD3^+^CD4^+^CD25^hi^CD127^lo^Foxp3^+^ T_reg_ cells for all fresh cord blood experiments.

### Stillbirth cohort

To determine any tissue specific alterations, we identified thymic, splenic, and lung tissue samples derived from stillbirths of varying gestational ages, with and without chorioamnionitis. The purpose of this cohort was to identify any tissue specific alterations in immune cell phenotype in chorioamnionitis.

We previously collected these samples as per hospital procedure, along with family consent for their use in research. We excluded samples from multiple gestations with umbilical cords that could not be designated, demises secondary to a primary immunodeficiency, subjects with known congenital/cytogenetic abnormality, and subjects without available lung or placental tissue. We identified 10 cases with and 10 cases without chorioamnionitis. One exposed subject did not have adequate splenic tissue present on autopsy; two control subjects did not have adequate thymic tissue. We present demographic data for this group in Table 2.

**Table 2:** Demographics pertaining to subjects that underwent tissue specific analyses

	Chorioamnionitis (n = 10)	Control (n = 10)
Maternal age (years)		
Average (range)	31 (23–40)	30 (21–38)
Median (interquartile range)	23 (21–24)	31 (26–35)
Race		
White	5	3
Black	5	3
Hispanic	0	2
Asian	0	1
Other	0	1
Sex		
Male	5	4
Gestational age		
Mean (range)	25.4 (18–40)	23.8 (19–34)
Median (interquartile range)	23 (21.3–24)	22 (20.3–23.8)

We stained thymus, spleen, and lung samples by immunohistochemistry for CD3 (DAKO/A0452), CD20 (DAKO/M0755) and Foxp3 (ABCAM/AB20034). We used cellSens^®^ (Lombard, IL, USA) image analysis software and assessed 10 digital image fields of each tissue for each case. The results were expressed as a mean percentage of the field occupied by the positive staining. We did not complete RORγt quantification because of technical problems with the staining.

### Statistical analyses

#### Cord blood analyses

We performed linear regression analysis to determine the association of RORγt^+^ expression in the Foxp3^+^ cells (CD3^+^CD4^+^Foxp3^+^) and T_reg_ cells (CD3^+^CD4^+^CD25^+^CD127^lo^Foxp3^+^) in individuals with chronic inflammation only (no fetal acute chorioamnionitis) compared to controls without acute high stage or chronic inflammation. We carried out similar regressions evaluating RORγt^+^ expression in the Foxp3^+^ cells (CD3^+^CD4^+^Foxp3^+^) and T-regulatory cells (CD3^+^CD4^+^CD25^+^CD127^lo^Foxp3^+^) in individuals with maternal inflammation only compared to subjects without acute high stage or chronic inflammation. We repeated this analysis comparing subjects with fetal inflammation to subjects without acute high stage or chronic inflammation. Given the low numbers, we presented the unadjusted results to avoid over-adjustment.

#### Tissue analyses

To account for correlations due to repeated measures, we performed a linear mixed model analysis to evaluate for any associations between chorioamnionitis and CD20, CD3, and Foxp3 expression. Due to skewness of all markers, we used a logarithmic scale for level of expression of CD20, CD3, and Foxp3 in our analysis. We used a secondary non-parametric analysis to account for the data non-normality. Due to variation in tissue composition and expected lymphoid cell density, we ranked the samples by cell number and retained the five images with the largest areas stained to be used as representative of the whole. We then used the median percent area stained of those images from the subjects to perform the nonparametric Wilcoxon rank sum tests.

We used STATA SE version 11 (College Station, TX, USA) to perform all analyses. The institutional review boards of the Ann and Robert H. Lurie Children’s Hospital of Chicago, and Northwestern University Feinberg School of Medicine approved the study.

## Results

### Live birth cohort

We found a significantly increased proportion of RORγt^+^ cells within the Foxp3^+^ cells (CD3^+^CD4^+^Foxp3^+^RORγt^+^) among individuals with chronic inflammation (no high stage acute inflammation) (β ± SE: 14.19 ± 3.59, *p* = 0.002; Table 3) when compared to individuals with neither acute or chronic inflammation. A sensitivity analysis in all individuals with chronic inflammation (regardless of the presence of acute inflammation, n = 19) also revealed significantly increased proportions of RORγt^+^ in the Foxp3^+^ cells (CD3^+^CD4^+^Foxp3^+^RORγt^+^) compared to those with no acute or chronic inflammation (β ± SE: 14.18 ± 3.59, *p* = 0.002). Interestingly, we found no statistically significant difference (β: − 1.09, SE: 2.04, *p *= 0.54; Table 3) in RORγt^+^ expression in CD3^+^CD4^+^CD25^+^CD127^lo^Foxp3^+^ cells between subjects with acute high-grade maternal inflammation and those without any inflammation.

**Table 3:** Frequency of RORγt^+^ cells in cord blood, organized by site of maternal and/or fetal inflammation and cell type

Cell type	Chronic inflammation with no acute inflammation (n = 8/15) β ± SE	p-value
RORγt^+^ in CD3^+^CD4^+^Foxp3^+^	*14.19 ± 3.59*	*0.002*
RORγt^+^ in CD3^+^CD4^+^CD25^+^CD127^lo^Foxp3^+^	− 1.10 ± 2.04	0.6

Cell type	Maternal high stage acute inflammation (stage 2 and 3) (n = 2/9) β ± SE	p-value
RORγt^+^ in CD3^+^CD4^+^Foxp3^+^	4.30 ± 4.30	0.35
RORγt^+^ in CD3^+^CD4^+^CD25^+^CD127^lo^Foxp3^+^	− 3.97 ± 6.09	0.54

Cell type	Fetal and maternal high stage inflammation (stage 2 and 3) (n = 2/9) β ± SE	p-value
RORγt^+^ in CD3^+^CD4^+^Foxp3^+^	*35.50 ± 3.80*	*< 0.001*
RORγt^+^ in CD3^+^CD4^+^CD25^+^CD127^lo^Foxp3^+^	− 1.80 ± 4.28	0.68

Note that there is a significant difference in RORγt^+^ cells in activated T cells compared in individuals with chronic inflammation with no acute inflammation compared to those with neither acute or chronic inflammation (p = 0.002). Also note the increase in RORγt^+^ cells in activated T cells in subjects with maternal and fetal high stage inflammation (p < 0.001), but not in subjects with maternal high stage inflammation alone

In addition, we found significantly increased RORγt^+^ cells within the Foxp3^+^ cells (CD3^+^CD4^+^Foxp3^+^RORγt^+^) in individuals with acute high stage fetal and maternal inflammation compared to those without acute or chronic inflammation (β: 35.50, SE: 3.80, *p* < 0.001; Table 3). Again, there was no statistically significant difference (β: − 1.80, SE: 4.28, *p *= 0.68; Table 3) in RORγt expression in CD3^+^CD4^+^CD25^+^CD127^lo^ Foxp3^+^ cells in these infants.

When evaluating the small number of subjects with maternal high stage acute inflammation, there was no statistically significant difference in the amount of Foxp3 expression on a per cell basis by mean fluorescence intensity (MFI) between neonates with maternal acute inflammation only compared to neonates without acute or chronic inflammation.

We next sought to determine the relationship between the percentage of RORyt^+^ in FOXp3^+^ compartment and premature rupture of membranes. A logistic regression analysis showed no difference between the percentage of RORyt^+^ in Foxp3^+^ compartment in those subjects that had premature rupture of membranes and those that did not.

### Stillbirth cohort

**Fig. 2:**
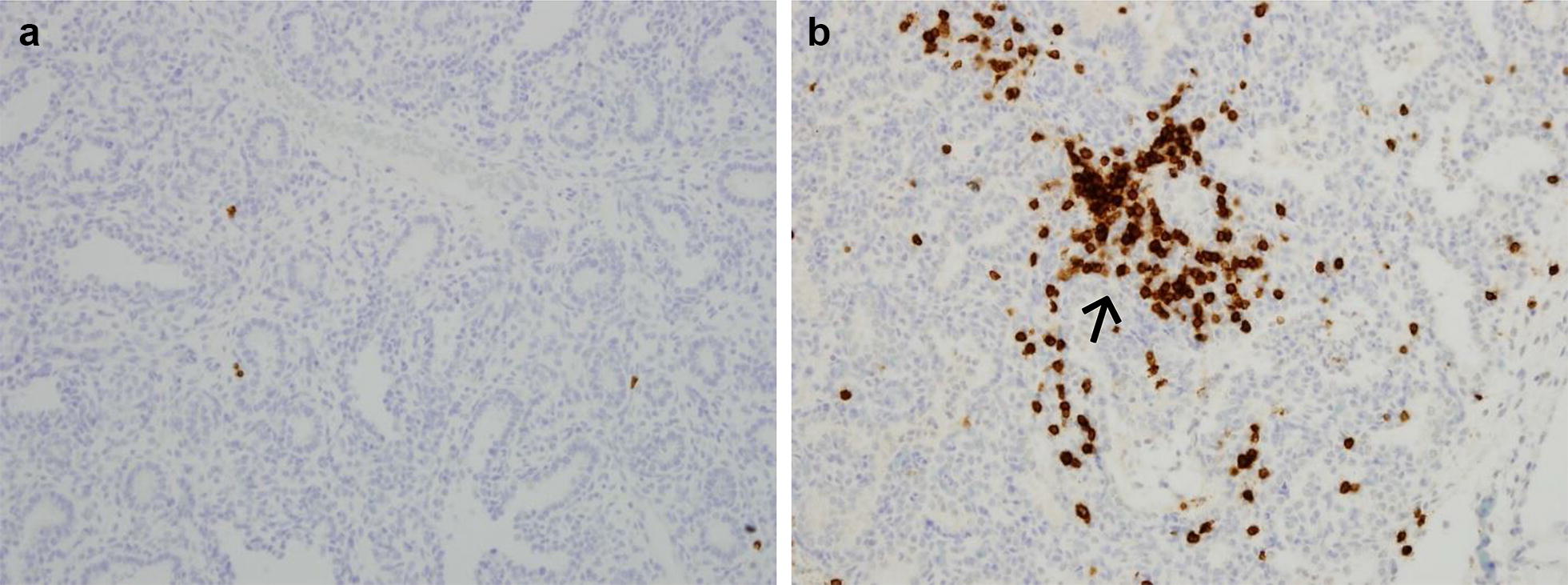
Stained tissue samples in individuals with and without chorioamnionitis. **a** CD3 staining of lung in subject without chorioamnionitis. **b** CD3 staining of lung in subject with chorioamnionitis. Note the increased CD3 stained lung tissue (indicated by the ➔) in the subject with chorioamnionitis (**b**) compared to the subject without the disease (**a**)

**Fig. 3:**
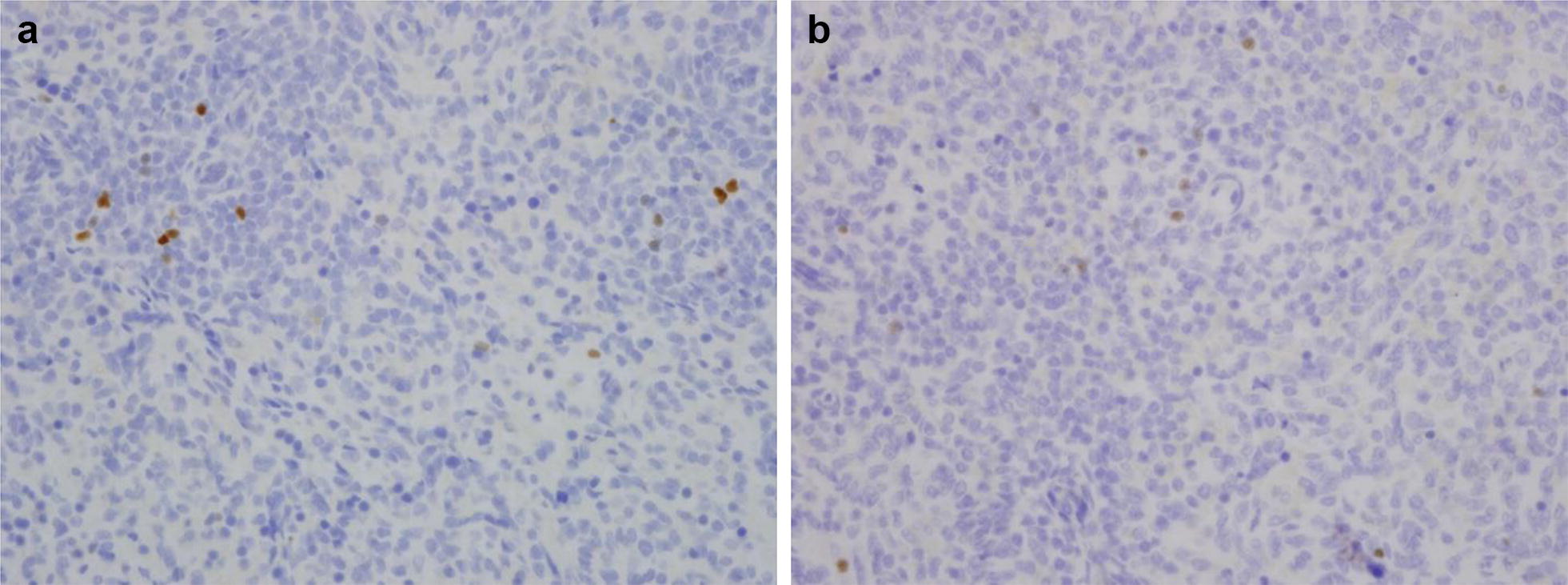
Stained spleen tissue samples in individuals with and without chorioamnionitis. **a** Foxp3 staining of spleen in subject without chorioamnionitis. **b** Foxp3 staining of spleen in subject with chorioamnionitis. Note there is no histologic difference in splenic FoxP3 stained tissue between the two subjects

Fetal splenic tissue showed significantly decreased Foxp3 staining (β: − 0.11, SD: 0.04, *p *= 0.01; Table 4) in subjects with acute chorioamnionitis compared to controls. Lung and thymic tissue showed no differences in Foxp3 staining between subjects and controls. Importantly, we found significantly increased CD3 staining in lung tissue in deceased subjects exposed to acute chorioamnionitis (β: 0.38, SD: 0.17, *p *= 0.05; Table 4) compared with controls. We found no differences in thymic or splenic CD3 staining in subjects with acute chorioamnionitis. We also found no differences in any tissue’s CD20 staining individuals with or without chorioamnionitis. Figures 2 and 3 show the representative staining for Foxp3 and CD3 in subjects with and without chorioamnionitis.

**Table 4:** Expression of CD20, CD3, and Foxp3 within fetal lung, splenic, and thymic tissue after chorioamnionitis

	Lung			Spleen			Thymus		
	Chorioamnionitis	Control	p-value	Chorioamnionitis	Control	p-value	Chorioamnionitis	Control	p-value
CD20 Median % (SD)	0.41% (0.03%)	0.13% (0.02%)	0.12	14.98% (0.56%)	11.66% (0.09%)	0.96	4.49% (0.03%)	2.31% (0.05%)	0.27
CD3 Median % (SD)	*0.53% (0.02%)*	*0.34% (0.003%)*	*0.05*	3.94% (0.044%)	5.49% (0.06%)	0.14	81.80% (0.11%)	79.29% (0.098%)	0.74
FoxP3 Median % (SD)	0.03% (0.0003%)	0.01% (0.0001%)	0.17	*0.00% (0.0004%)*	*0.09% (0.002%)*	*0.01*	0.06% (0.004%)	0.37% (0.006%)	0.17

Data expressed as median (standard deviation). Note significantly increased CD3 expression in fetal lung and significantly decreased Foxp3 expression in the fetal spleen in subjects with chorioamnionitis

Lastly, a secondary non-parametric analysis, to account for the data non-normality using the median percent area stained from each subject, yielded the same findings as the linear mixed model analysis.

## Discussion

This investigation provides an initial immunologic characterization of human cord blood and tissue responses to acute high stage placental inflammation and chronic placental inflammation. We found a significantly increased frequency of cord blood RORγt^+^Foxp3^+^ cells in individuals with both chronic placental inflammation and high stage acute fetal inflammatory response in the placenta. Moreover, we found that fetal tissue responses to histologic acute chorioamnionitis revealed a decreased percentage of splenic Foxp3^+^ cells in the stillbirth subjects. We also found an increase in percentage lung CD3^+^ cells in stillbirth subjects that had chorioamnionitis compared with controls. We believe that these findings suggest that decreased Foxp3^+^ cells in the spleen in acute chorioamnionitis may be associated with a decreased T-regulatory component and concomitant upregulation of activated T-cells in the lung. It is possible, but not yet proven, that these changes may contribute to a proinflammatory environment in the lung and confer this increased asthma risk seen in these patients.

Animal models have demonstrated plasticity between T_reg_ or T_H_17 cells after exposure to specific cytokines [20, 21]. Foxp3 can be transiently upregulated with inflammation in the human. Thus, it is possible that the Foxp3^+^RORγt^+^ cells represent either activated T_H_17 cells or regulatory T cells that are now expressing a pro-inflammatory phenotype. Additionally, “regulatory Th-17 cells” that express both Foxp3 and RORγt have been described that have immunosuppressive properties in the gut. These cells likely promoting tolerance to the microbiota. It is likely that the context of Foxp3^+^RORγt^+^ cells determine if they are pathogenic (for example in setting of inflammation) or tolerogenic (microbiota) [15]. Given this duality of function, it is not clear if the cells we describe are truly pathogenic vs tolerogenic; however, we suspect they are pro-inflammatory given the setting of inflammation in chorioamnionitis and that they were not isolated from the gut.

Although Foxp3 expression can block RORγt activity on target genes during T_H_17 cell differentiation RORγt-dependent IL-17 expression can still occur in the presence of Foxp3 with IL-6 exposure [21, 22]. Because the cytokine profile in acute chorioamnionitis includes significant increases in IL-6, we speculate that these increases in IL-6 contribute to elevation of RORγt^+^ cells seen in our analysis. Furthermore, work from non-human primate models have shown increased T_H_17 cells in a chorioamnionitis fetal inflammatory response [23]. The exposed primates also showed decreased thymic and splenic T_reg_ frequency [23]. These findings, similar to our own, suggest a transition from regulatory to inflammatory phenotype in response to chorioamnionitis.

The lack of difference in T-cell numbers in samples with maternal acute inflammatory response alone in the placenta compared to samples with controls might be accounted for by the small numbers of subjects in the sample or the severity of inflammation in the fetus. Maternal and fetal acute inflammatory responses in the placenta may promote more widespread/systemic inflammation within the fetus, leading to the increased T_H_17 cells. It is unknown whether increased RORγt expression may then prime individuals to have a persistent pro-inflammatory state, and whether this pathway explains the increased risk of subsequent asthma in childhood [1, 2]. Notably, the subjects with high stage fetal acute inflammation also had chronic placental inflammation, making it not possible to control for the effects of chronic placental inflammation due to collinearity. However, we note the effects of fetal inflammation are much greater than those seen in chronic inflammation by itself. When we restricted the analysis to those who had chronic inflammation but no chorioamnionitis, these subjects showed similar responses, though smaller in magnitude, to those with fetal inflammation. This suggests that chronic low-grade inflammation may result in similar though less pronounced immune deviation. This distinction is important to make because acute and chronic chorioamnionitis represent different pathological entities with acute chorioamnionitis manifested by neutrophilic inflammation and typically associated with acute infection [24, 25]. Chronic chorioamnionitis is a lymphocytic infiltrate which includes villitis of unknown etiology and may have some associated inflammatory or alloimmune factors [26]. Animal models to date have been carried out with LPS injection resulting in massive and acute increases in IL-1b and IL-6 [17, 18]. It was not clear that chronic inflammatory lymphocytic changes would have similar levels of inflammatory cytokine expression. As such, it was important to determine if the systemic and organ responses are the same in acute and chronic inflammation.

In addition to animal models, Jackson et al. investigated the association between chorioamnionitis exposure and the cellular profile from cord blood and weekly for the first 4 weeks of life in extremely preterm infants (≤ 28 weeks gestation) [27]. They analyzed mRNA expression of *RORC*, *TBET*, *GATA3*, and *FOXP3* in unstimulated cells and several inflammatory cytokines [27]. They found that at 1 week of life infants, with funisitis exposure and not chorioamnionitis exposure had increased RORC expression and *RORC*/*FOXP3* ratio, which continued over the first 4 postnatal weeks [27]. Our current study adds to these findings by suggesting differential cellular RORγt expression in infants exposed to chorioamnionitis, suggesting a skewing towards a T_H_17 phenotype after fetal stress. In addition, while Jackson et al. used mRNA analysis, our study investigated cell marker protein expression, which lends additional insight. Furthermore, while Jackson et al. investigated the association between chorioamnionitis/funisitis on preterm infants, our study used term infants [27]. Lastly, our investigation analyzed tissue differences in cell expression in addition to cord blood differences, which has not been previously studied in humans.

We also noted increased percentage of pulmonary CD3^+^ T-cells and decreased splenic Foxp3^+^ T-cells with acute chorioamnionitis. The increase in CD3^+^ T-cells in the lung and decrease in Foxp3^+^ expressing cells in the splenic tissue may indicate localized pulmonary increases in inflammation with decreased regulatory responses systemically. Importantly, this early life inflammatory predisposition in the airway may be integral in increasing the risk for wheezing and asthma seen in these patients. Supporting this notion, animal models have shown an increased splenic CD3^+^ cells after exposure to lipopolysaccharide seen in intrauterine inflammation [7]. In our study, we found fewer Foxp3 cells in the spleen in infants with chorioamnionitis. This decrease in Foxp3 cells may be an effect of the proinflammatory local environment. Lastly, it is known that IL-6 and TNF-alpha are increased in chorioamnionitis and these cytokines also result in methylation at the Foxp3 promoter, that then down regulates T_reg_ cell development [20, 28–32].

Our study does have several limitations. First, the number of subjects with histology acute chorioamnionitis was small in the live birth cohort. This may bias our findings and limit the generalizability based on the limited sample. A larger sample size would ultimately help with this in future studies in order to determine if similar effects are seen in individuals with chorioamnionitis compared with controls. Secondly, in the stillbirth cohort, we could not evaluate RORγt in tissues. Due to technical issues, this analysis was not performed, which ultimately hinders our findings. If RORγt had been evaluated in tissues, we would be able to better determine if the increased T_H_17 profile seen in the cord blood persisted in fetal tissues. We anticipate investigating for tissue T_H_17 cells in future studies. Furthermore, we did not evaluate the stillborn infants’ cord blood. Investigating cord blood in these infants would have been the best way to evaluate any association between systemic and tissue responses to chorioamnionitis by the neonates. We also anticipate performing these in future studies. Due to findings in animal models, we focused on T_H_17 and T_reg_ phenotypes with less investigation of other cell types, which is also a limitation [17–19]. Therefore, we would hope to investigate other cell types in addition to T_H_17 and T_reg_ cells in future studies. In addition, further investigation including expression studies may show tissue-specific increases of activated T_H_17 cells. Finally, we believe that future investigation should determine whether the observed RORyt cells are αβ or γδ CD4^+^ T-cells.

In summary, the presented data supports that hypothesis that fetal inflammation promotes immune deviation seen in patients with chronic placental inflammation or acute chorioamnionitis with a fetal inflammatory response. This immune deviation may ultimately play an early role in predisposition to asthma that we observe in infants who had chorioamnionitis. We view our results as a key step into understanding this link between chorioamnionitis and asthma. We hope that future studies investigate whether elevations in T_H_17 cell frequency persist into childhood, and how these may relate to later asthma phenotypes.

## Additional file

**Additional file 1: Figure S1.** Venn diagram of the number of patients with each combination of inflammation in the live-birth cohort. Note that there were no subjects with only fetal high stage acute inflammation and no subjects with fetal high stage acute inflammation along with chronic inflammation.

## Abbreviations

IL: interleukin
T_H_17: T-helper-cell 17
T_reg_: regulatory T-cell
RORγt: retinoic acid-related orphan receptor gamma t
MFI: mean fluorescence intensity
FMO: Fluorescence Minus One
H&E: hematoxylin and eosin
