# Supplementary material for: Fetal cord blood and tissue immune responses to chronic placental inflammation and chorioamnionitis
Source: Allergy Asthma Clin Immunol. 2018 Nov 19;14:66. doi: 10.1186/s13223-018-0297-y (PMC6240933; doi:10.1186/s13223-018-0297-y)

Figure S1: Venn diagram of the number of patients with each combination of inflammation in the live-birth cohort. Note that there were no subjects with only fetal high stage acute inflammation and no subjects with fetal high stage acute inflammation along with chronic inflammation.

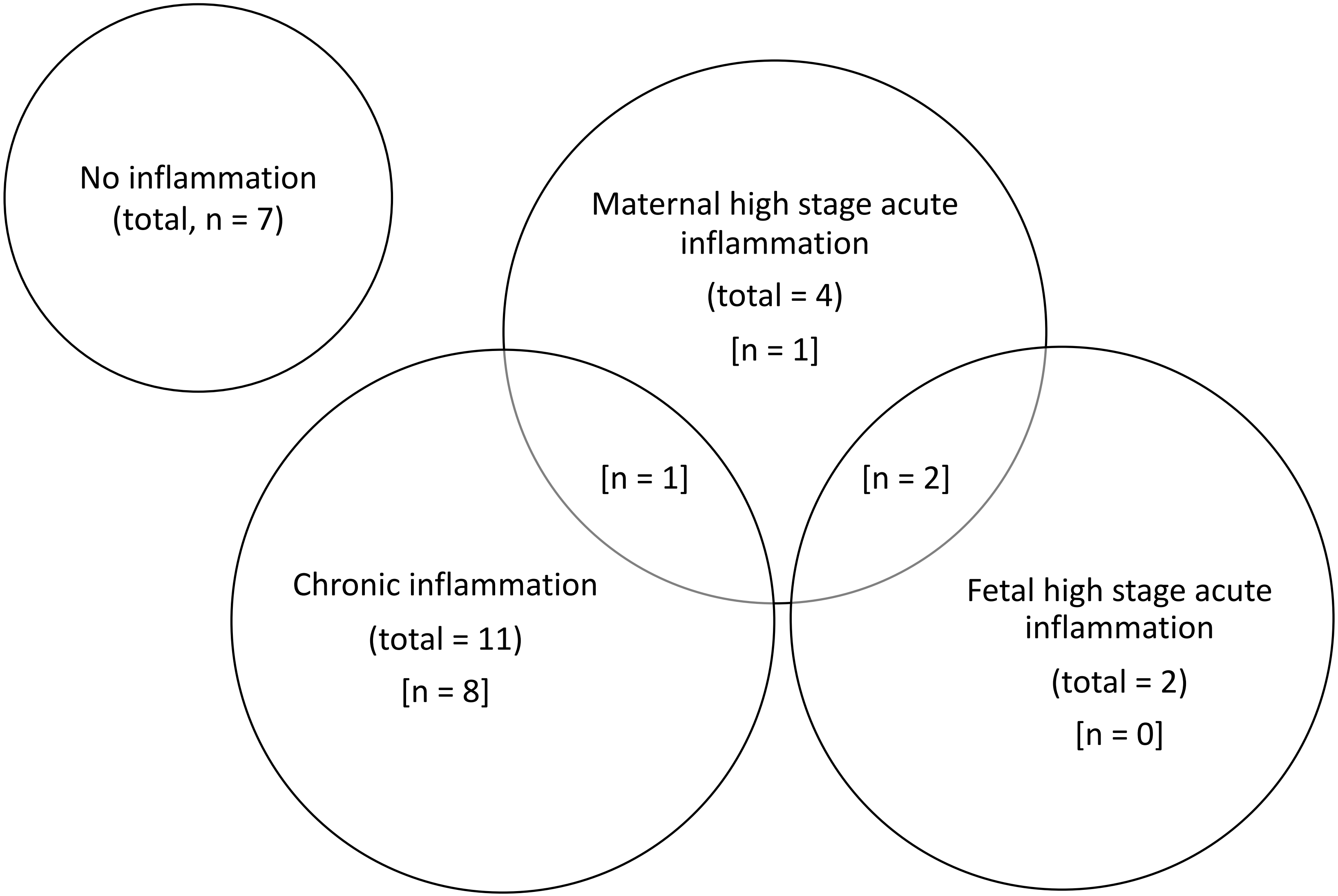

Supplement: Supplementary file 1 — Additional file 1: Figure S1. Venn diagram of the number of patients with each combination of inflammation in the live-birth cohort. Note that there were no subjects with only fetal high stage acute inflammation and no subjects with fetal high stage acute inflammation along with chronic inflammation. [file 13223_2018_297_MOESM1_ESM.pdf]
